# Psychometric properties of the Persian translation of maternal postpartum quality of life questionnaire (MAPP-QOL)

**DOI:** 10.1186/s12955-021-01781-1

**Published:** 2021-05-08

**Authors:** Tahereh Mokhtaryan-Gilani, Giti Ozgoli, Nourossadat Kariman, Hamid Sharif Nia, Mahbobeh Ahmadi Doulabi, Malihe Nasiri

**Affiliations:** 1 Midwifery and Reproductive Health Department, School of Nursing and Midwifery, Shahid Beheshti University of Medical Sciences, Tehran, Islamic Republic of Iran; 2 Midwifery and Reproductive Health Research Center, Midwifery and Reproductive Health Department, School of Nursing and Midwifery, Shahid Beheshti University of Medical Sciences, Vali-Asr Avenue, Vali-Asr and Neiaiesh Highway Intersection, Opposite Rajaee Heart Hospital, P.O. Box: 1996835119, Tehran, Islamic Republic of Iran; 3 School of Nursing and Midwifery Amol, Mazandaran University of Medical Sciences, Sari, Iran; 4 School of Nursing and Midwifery, Shahid Beheshti University of Medical Sciences, Tehran, Islamic Republic of Iran

**Keywords:** Quality of life, Postpartum, Questionnaire

## Abstract

**Background and objective:**

Many studies have been conducted in Iran on the postpartum quality of life, and the majority have used the general quality of life questionnaire. With a specific tool in this context, the dimensions of maternal postpartum quality of life can be more accurately determined. The present study was conducted to determine psychometric properties and validate the Persian version of the Maternal Postpartum Quality of Life Questionnaire (MAPP-QOL).

**Materials and methods:**

The present methodological study was conducted in 2018. The original version of MAPP-QOL was translated into Persian by both forward and backward translation. In a cross-sectional study, the Persian version was completed by 407 eligible postpartum women aged 18 to 47 and living in Tehran. Reliability of the questionnaire was assessed using Cronbach's alpha coefficient and test–retest. For construct validity, exploratory and confirmatory factor analyses were used.

**Results:**

The MAPP-QOL showed good content validity; content validity ratio (CVR) ranged from 0.6 to 1.00, and content validity index (CVI) ranged from 0.7 to 1.00. Using exploratory factor analysis, five factors, including Socioeconomic; Relational/Family-Friends; Psychological/Baby; Health & functioning; and Relational/Spouse-Partner, were extracted, which together explained 78.84% of the total variance. After modifications of CFA, the confirmatory factor analysis showed an acceptable goodness-of-fit. AVE value Above 0.5 exhibited appropriate convergent validity, and AVE greater than MSV confirmed divergent validity. The Cronbach's alpha, McDonald's Omega, Composite reliability and maximum reliability H of the five extracted factors were excellent (> 0.9). Also, the AIC values of the factors were good (between 0.721 to 0.859).

**Conclusion:**

The 38-item Persian version of the postpartum quality of life questionnaire is adequately reliable for postpartum women in Iran. Given its appropriate psychometric properties, this scale is fit to be used in future studies on postpartum women.

## Introduction

Quality Of Life (QOL) is the individuals' understanding of their situation in life according to their culture, value, attitude, goals, expectations and standards [1], which has been used in many studies in the field of health cares [2].

Measurement of QOL has been developed in the last 30 years and has become a formal discipline with specific theoretical and methodological structural bases [3]. Thus, QOL has been increasingly recognised as an essential standard of outcome in studies on treatments, and evaluation of services [4]. Accordingly, the evaluation of postpartum QOL is essential for health promotion planning [5].

The word postoartum is derived from Latin—puer, child and parus, bringing forth. Currently, it defines the time following delivery during which pregnancy-induced maternal anatomical and physiological changes return to the nonpregnant state. Its duration is understandably inexact, but is considered to be between 4 and 6 weeks[6]. In fact, no event like childbirth requires rapid changes in the mother's body to become compatible with the maternal role. Also, quality of life is affected as one of the indicators following childbirth [7, 8].

Postpartum is a critical period for a woman in which increasing complications and impaired quality of life may appear. As a mother experiences physical and psychological conditions changes during postpartum, additional responsibilities of providing neonatal cares as well as her role in the family, inadequate sleep and fatigue make her spend less time on herself, which leads to demoralisation, and reduced quality of life [9, 10].

Very few tools have been designed to evaluate postpartum QOL, one of which is the Mother-Generated Index (MGI), an open subjective self-administered scale. Even with investigators' help, participants still need to have sufficient cognitive skills to understand the questions well and write informative answers because postpartum women's emotional state might easily influence the answers. These shortcomings have restricted the use of this scale, indicating that it cannot be an appropriate questionnaire to evaluate the postpartum QOL [11].

PQOL is a Chinese Postpartum QOL questionnaire that appears to be compatible with Chinese women's culture and probably needs to be further developed by assessing its psychometric properties in other countries [10].

The majority of studies conducted in Iran on postpartum quality of life have used general quality of life tools. In fact, the need for a specific Persian questionnaire with acceptable validity and reliability is being felt. That is why the present study was conducted to translate MAPP-QOL and assess its psychometric properties in Iran. MAPP-QOL had more specific dimensions than other specific postpartum quality of life instruments.

The purpose of this study is to evaluate the psychometric properties of the Persian translation of Maternal Postpartum Quality of Life Questionnaire (MAPP-QOL).

## Materials and methods

The present methodological study was conducted in 2018 on women living in the city of Tehran. At first, the city of Tehran was divided into five regions. Two health centres with the highest number of clients were selected from each region. Eligible individuals were chosen by purposive and convenience sampling method from each centre. Then, after obtaining written consents, the digital questionnaires were sent to them online.

Four hundred and seven 18 to 47 years old mothers who went to health centres to get their babies vaccinated and checked up, were recruited within 1 to 3 weeks after their childbirth (207 and 200 participants separately for exploratory and confirmatory factor analysis respectively). According to the inclusion criteria, the mothers of live babies, who understood the Persian language and were able to read, and signed the written consent form, were included. Based on the exclusion criteria, physically or mentally disabled women and those with severe chronic diseases were excluded. For example, those with mental retardation, physical disability, high-grade and debilitating heart disease, and advanced cancers were among the study's excluded ones.

Data were collected using a three-part questionnaire consisting of a demographic part (based on education, age and occupation), a clinical characteristics part (gestational age, gravidity, type of infant feeding, type of delivery and maternal complications), and a MAPP-QOL part.

Psychometric properties of MAPP-QOL were assessed by Hill et al. [12] in America on 184 women in their first and third postpartum weeks. In their study, tool-making was carried out using France's conceptual model of Quality of Life Index (QLI) and its dimensions [12]. The questionnaire designer was contacted by email, and his permission for the present study to translate the questionnaire and determine its psychometric properties was obtained.

In the second stage, the MAPP-QOL questionnaire was translated according to the protocol declared by WHO and established by Harvard Medical School [13]. The translation process consisted of the following steps:

### Forward translation

An Iranian reproductive health professional alongside an expert translator performed the translation of the original version of the MAPP-QOL to Persian. Instructions were given to focus on a conceptual rather than a literal translation, and use plain language to support the general public.

### Expert panel

Five reproductive health professionals, who were experts in this study's methodology, the occupational field of reproductive health, and the translation of the first version of the questionnaire, were selected to perform this part. After the panel members' evaluation, all notes were reviewed, and MAPP-QOL version 1 was produced.

### Back-translation

The MAPP-QOL version 1 was back-translated into English by an independent English translator, unfamiliar with the original questionnaire. This version was sent to the questionnaire author for approval (version 2).

### Pretesting and cognitive interviewing

During the pretest, the time that the professionals dispensed to answer the questionnaire were scrolled. Then, we asked them about the understanding of each statement, the given response, and suggestions to make the questionnaire more understandable and easier to apply. During the pretesting part, each sample print was carefully considered for preparing the 3rd version of the MAPP-QOL.

Ten postpartum women aged between 20 and 39 years were interviewed. Based on the instrument, all the women answered the questionnaire individually. The time used by the participants to complete the questionnaire ranged from 25 to 30 min.

While performing this part, participants were asked to opine on each item and the questionnaire in general. Participants were unanimous in considering the questionnaire easy to understand.

The new translated English version was sent to the author of the original Mapp QOL, and modifications were made according to the author's views(version 4).

### Final version

The final Persian questionnaire was the result of the procedure described above.

The validity and Reliability of MAPP-QOL and its psychometric properties were assessed as follows:

#### Face validity

Face validity was evaluated for relevancy. In the qualitative evaluation, the Persian version of the questionnaire was given to 20 postpartum women of different socioeconomic status. The views expressed by the target group (i.e. postpartum women) about the level of difficulty, relevancy and ambiguity of the items were considered. The necessary modifications were made to the items to make them easily comprehensible. The items' impact scores were calculated to eliminate the inappropriate items and determine each item's importance regarding the quantitative method. Thus Items with an impact score > 1.5 were retained for further analysis.

#### Content validity

Ten experts who were experienced in the fields of quality of life, postpartum period, and instrument design were referred to in order to determine the satisfactions of qualitative and quantitative validity. The qualitative evaluating in terms of grammar, use of the right words, placement of the items, proper scoring, and modifications were applied based on their feedbacks. Also, the Content Validity Ratio (CVR) and Content Validity Index (CVI) were measured for the quantitative content validity assessment. In this study, the Content Validity Ratio (CVR) was determined based on the experts' views, who were asked to divide items into three categories, including "Essential", "Useful but not essential", and "Not essential", and the CVR was then calculated using the following formula:

$$CVR = \frac{{N_{e} - \frac{N}{2}}}{\frac{N}{2}}$$

Ne is the number of experts who have considered an item essential, and N is the total number of experts in the study.

#### Construct validity assessment

The construct validity of MAPP-QOL has been assessed through the Maximum-Likelihood Exploratory Factor Analysis (MLEFA) method with Promax rotation. Sample adequacy was estimated through the Kaiser–Meyer–Olkin (KMO) and Bartlett's tests. KMO values of 0.7–0.8 and 0.8–0.9 were interpreted as good and excellent, respectively. The sample size for factor analysis was estimated by the rule of thumb, which considers 200 participants adequate [14]. Thus, 207 participants were recruited for Exploratory Factor Analysis (EFA) and 200 for Confirmatory Factor Analysis (CFA). A purposive and convenience sampling of newly delivered mothers who attended ten medical centres in Tehran (Iran) was approached.

The presence of each item in a latent factor was determined based on a factor loading of more than 0.3, which was estimated using the following formula: *CV* = *5.152* ÷ *√ (n* − *2)*, where *CV* was the number of extractable factors and *n* was the sample size [15]. The number of latent factors was estimated using Horn's parallel analysis [16]. Next, items with communalities less than 0.2 were excluded from EFA [17]. CFA was conducted to assess the structural factors using the maximum-likelihood method and the most common goodness of fit indices. The model fitness was assessed according to Root Mean Square of Error of Approximation (RMSEA), Comparative Fit Index (CFI), Parsimonious Normal Fit Index (PNFI), Incremental Fit Index (IFI), and Parsimonious Comparative Fit Index (PCFI).

#### Convergent and divergent validity assessment

The convergent and divergent validity of MAPP-QOL were estimated using Fornell and Larcker's approach and through the Average Variance Extracted (AVE), the Maximum Shared Squared Variance (MSV) and Composite Reliability (CR).

#### Reliability assessment

The internal consistency of MAPP-QOL was assessed via calculating Cronbach's alpha, McDonald's omega and average inter-item correlation (AIC) [18]. Cronbach's alpha and McDonald's omega value of more than 0.7 and AIC value of 0.2–0.4 were considered acceptable [18]. CR is a substitute for Cronbach's alpha in structural equation modelling. CR of more than 0.7 is acceptable. Finally, Maximum Reliability H was calculated. The H values greater than 0.8 were also exceeded as an item [19].

#### Normal distribution of the data, outliers, and missing data

At first, the data's univariate distribution was estimated using the skewness and the kurtosis, while the multivariate distribution was assessed using the Mardia coefficient. Moreover, the existence of multivariate outliers was assessed through Mahalanobis distance. There were no missing data because participants filled out the online questionnaire. Univariate distributions were examined for the outliers. Multivariate distributions were evaluated for normality and multivariate outliers [18]. Multivariate normality can be evaluated through the use of the Mardia's coefficient of multivariate kurtosis. A Mardia's coefficient of more than 8 indicated deviation from the normal distribution [20]. The Mahalanobis distance items smaller than 0.001 were considered multivariate outliers [21].All data analyses were performed using the SPSS-AMOS_24_, JASP_0.11.1_ and the SPSS R-Menu_2.0_.

#### Ethical considerations

Permission was first obtained from the questionnaire designer for the translation and psychometric assessment. After introducing the study, some eligible women in the postpartum period voluntarily filled the MAPP-QOL questionnaire. The study objectives were explained to them, and participants were assured of the confidentiality of all data.

## Results

Four hundred and seven 18 to 47 years old mothers were recruited within 1 to 3 weeks after their childbirth (207 and 200 participants separately for exploratory and confirmatory factor analysis respectively).

Once the qualitative face validity was assessed, one item (item 17) was modified based on the women’ views, because some mothers did not have a proper understanding of the concept of partner. In the quantitative validity assessment stage, all the items had impact scores > 1.5 (ranging from 2.3 to 4.7) and were therefore retained. The views expressed by the professors and experts were implemented after the qualitative content validity assessment stage. Lawshe's method was used to determine the quantitative content validity of the instrument. In the present study, good content validity was showed; content validity ratio (CVR) ranged from 0.6 to 1.00, and content validity index (CVI) ranged from 0.7 to 1.00

For Exploratory factors analysis, 207 of participants aged between 18 and 47 years, with a mean age of 24.54 ± 3.55 years, gestational age of 38.40 ± 2.42 weeks, and parity of 1.33 ± 0.53 infants born (48.3% girls and 51.7% boys), with 64.3% of vaginal childbirths and 35.7% cesareans were chosen. Of the infants born, 87% were breastfed, 12% were fed on powdered milk, and 1% on both. 69% of the women were housewives, 31% were employed, 57% had a university education, and the rest were high school or lower graduates.

The latent factors with Eigenvalues > 1 were extracted by EFA and varimax rotation.In MLEFA, KMO test, the value was 0.918, and in Bartlett's test, the value was 21,350.080 (*P* < 0.001). MLEFA revealed a five-factor structure for MAPP-QOL consisting of Socioeconomic, Relational/Family-Friends, Psychological/Baby, Health & functioning, and Relational/Spouse-Partner factors. After the analysis, 38 of the scale items were retained. These five factors' eigenvalues were 7.752, 6.868, 6.506, 5.148, and 3.453, respectively. 78.84% of the total variance of the MAPP-QOL were explained by these factors (Table 1).

**Table 1:** Exploratory factors extracted from items of MAPP-QOL

Factor	Q_n_. Item	Factor loading	h^2^*	%Variance	Eigenvalue
	How satisfied are you with:				
Socioeconomic	36. Your economic or financial capacity?	0.965	0.931	20.400	7.752
	35. Your materialistic possessions?	0.961	0.924		
	32. Your ability to meet financial?	0.960	0.925		
	31. Your financial independence?	0.919	0.828		
	38. Your husband’s employment?	0.907	0.812		
	29. Your home/apartment/place where you live?	0.861	0.749		
	33. Your access to medical care?	0.816	0.647		
	34. Your access to transportation?	0.810	0.644		
	30. Your neighborhood?	0.809	0.666		
	39. Your own employment?	0.769	0.654		
Relational/family-friends	22. Time for maintaining the household?	0.974	0.932	18.070	6.868
	24. Time for husband/partner?	0.973	0.933		
	18. Your ability to meet family responsibilities?	0.957	0.912		
	15. The emotional support you get from: your extended family?	0.936	0.865		
	23. Time for friends/relatives?	0.926	0.884		
	25. Time for yourself?	0.865	0.813		
	16. Your friends or other people?	0.858	0.751		
	20. The assistance with baby care and other children?	0.741	0.552		
	21. Time for children?	0.539	0.283		
Psychological/baby	12. Your life in general?	0.969	0.932	17.120	6.506
	11. Your happiness in general?	0.950	0.931		
	19. Your baby’s health?	0.938	0.768		
	10. Your peace of mind?	0.933	0.905		
	28. Your day-to-day life’s routine?	0.924	0.808		
	4. Amount of control you have over your life?	0.894	0.842		
	13. The amount of worries in your life?	0.825	0.754		
	26. Your ability to feed your new baby?	0.762	0.567		
Health and functioning	3. Amount of energy for everyday activities?	0.915	0.834	13.540	5.148
	8. Your surgical incision or episiotomy?	0.902	0.791		
	5. Your ability to take care of yourself without help?	0.893	0.825		
	7. Your breasts?	0.884	0.758		
	2. The amount of pain that you have?	0.876	0.765		
	9. Your sex life?	0.845	0.807		
	1. Your health?	0.665	0.397		
Relational/spouse-partner	14. The emotional support you get from: your husband/partner?	0.978	0.947	9.080	3.453
	17. Your relationship with your husband/partner?	0.966	0.935		
	27. Your husband/partner’s health?	0.919	0.832		
	37. Your overall environment /surroundings? (no yelling, fights, squabbles)	0.848	0.754		

^*^Communality

For confirmatory factor analysis, 200 participants aged between 18 and 47 years, with a mean age of 33.85 ± 3.50 years, gestational age of 38.29 ± 1.67 weeks, and parity of 2.11 ± 1.30 the infants born (43% girls and 57% boys), were chosen. 52.5% of childbirths were vaginal and 47.5% by cesarean. Of the infants born, 84.5% were breastfed, 14.5% were fed on powdered milk, and 1% on both. 62.5% of women were housewives, 37.5% were employed, 65% had a university education, and the rest were high school or lower graduates.

A Confirmatory Factor Analysis (CFA), which the results are presented in Table 2, was carried out first. A CFA was performed based on the results of this EFA. Table 2 presents the CFA results to compare with the results of the original scale.The goodness of fit of the 38-item questionnaire's final factor structure was assessed using the Chi-square goodness of fit test with CFA. Then, the other indices' model fit was assessed, and the results obtained confirmed the suitable fit of the model.

**Table 2:** The fit model indices of CFA of MAPP.QOL

Model	Indices*									
	χ^2^	df	*P* value	CMIN/DF	RMSEA	PCFI	PCFI	PNFI	IFI	CFI
First-order	1677.570	644	< 0.001	2.604	0.051	0.831	0.831	0.786	0.908	0.907

*Acceptable values are as follows: > 0.500 for PNFI, PCFI, AGFI; > 0.900 for CFI and IFI; > 0.080 for RMSEA; and > 0.500 for CMIN/DF

**Fig. 1:**
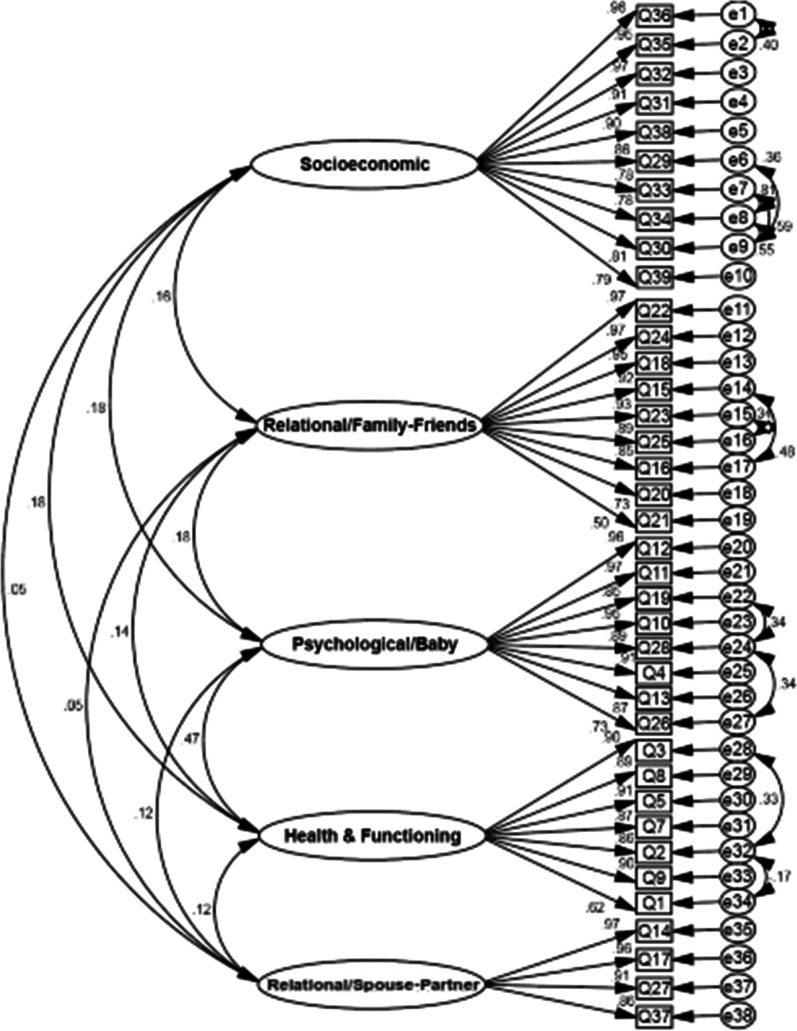
The final structural model of MAPP-QOL

After modifications of CFA, all goodness of fit indices confirmed the model fit (χ^2^ = 1677.570; N = 200; df = 644, *P* < 0.001; PCFI = 0.831; PNFI = 0.786; CMIN/DF = 2.604; RMSEA = 0.051; IFI = 0.908, CFI = 0.907) (Table 2 and Fig. 1).

Table 3 shows that the AVE factors' values were more than 0.5, indicating a good convergent validity for all factors. Also, the MSV values of the other factors were less than AVE, so the divergent validity was confirmed (Table 3 and Fig. 1).

**Table 3:** Internal consistency, convergent, divergent validity and composite reliability indices of the Persian MAPP-QOL

Factor	α	Ω	AIC	CR	AVE	MSV	MaxR(H)
Socioeconomic	0.973 (0.968–0.976)	0.973	0.785	0.970	0.766	0.034	0.982
Relational/family-friends	0.960 (0.954–0.965)	0.966	0.741	0.964	0.756	0.033	0.984
Psychological/Baby	0.969 (0.964–0.973)	0.971	0.805	0.970	0.802	0.223	0.982
Health and functioning	0.947 (0.939–0.955)	0.950	0.950	0.949	0.731	0.223	0.959
Relational/spouse-partner	0.960 (0.953–0.966)	0.961	0.859	0.961	0.860	0.015	0.975

*α* Cronbach’s alpha; *Ω* McDonald’s omega; *AIC* average inter correlation; *CR* composite reliability; *AVE* average variance extracted; *MSV* the maximum shared squared variance; *MaxR*(*H*) coefficient H

The Cronbach's alpha, McDonald's omega, CR and maximum reliability H of the five extracted factors were excellent (> 0.9). Also, the AIC values of factors were good (between 0.721 and 0.859) (Table 3).

## Discussion

The present study was conducted to assess the validity, reliability, and factor structure of the Persian version of MAPP-QOL, and present a reliable tool in the Persian language compatible with Iranian postpartum women's culture. All 407 of Participants were aged between 18 and 47 years, with mean age of 29.21 ± 5.68 years, end of pregnancy 38.34 ± 2.08 weeks, and parity 1.56 ± 0.89.

Validity and reliability are usually the key quality indicators of measurement instruments. In simple words, validity indicates the rigour and accuracy of a tool, and reliability shows its stability [22]. MAPP-QOL is specifically designed for women in postpartum and has been developed according to their views. All 38 items of 5 factors of the questionnaire were translated in a simple, clear and relevant manner, and the Persian version obtained an acceptable face and content validities.

The majority of the comments raised by the participants were clearly related to the experience of childbirth and motherhood (e.g., comments relevant with infant care, breastfeeding, sleep and fatigue, and relationships with family and husband). A large sample size increases the credibility of a study and its generalizability to a larger population. For the bulk of 38 items, the factor analysis supported the original five conceptual domains (Socioeconomic, Relational/Family-Friends, Psychological/Baby, Health & Functioning, and Relational/Spouse-Partner).

Internal consistency of the translated questionnaire in five subscales of MAPP-QOL with Cronbach's alpha of 0.907 and appropriate reliability indicate its measurement stability and compatibility of items with each other [23]. In the present study, the five extracted factors together explained 78.84% of the total test variance. In a study by Hill et al. [12] who designed the original version of this questionnaire with 39 items, (given that this construct had acceptable validity), internal consistency reliability in five subscales of MAPP-QOL had Cronbach's alpha of 0.96, and the five extracted factors together explained 59.7% of the total test variance (with the same five factors). In the present study, exploratory and confirmatory factor analyses were used, while the original version used exploratory and Spearman correlation coefficient. Both translated and original versions had favourable validity and reliability.

The designer of MAPP-QOL suggested that this study should be conducted for larger populations with diverse subjects [12]. In the resent study, we had a diverse population of all eligible ages, educational and cultural backgrounds.

Many studies have used general quality of life instruments to assess the quality of life after childbirth, while general instruments cannot accurately measure the mother's condition in certain circumstances and may lack some important dimensions in the postpartum period. Limited specific instruments are designed to measure the quality of life after childbirth precisely. Symon et al. designed the first dedicated scale of quality of life after childbirth (MGI) in 2001, which is an open subjective self-administered scale [21]. The difficult response, time-consuming, and complexity of this instrument's implementation by mothers have limited its widespread use [24]. Hill et al. designed the MAPP-QOL tool in 2006, and Zhou et al. designed the specific instrument of PQOL in 2009 to assess the quality of life after childbirth [12, 25]. The present study sampled more than 2 to 3 times of the above studies. For structural validity, in addition to heuristic factor analysis, parallel and confirmatory factor analysis were also used to evaluate the instrument, which has not been seen in the three mentioned previous studies. To assess the reliability of the current instrument, in addition to Cronbach's Alpha (0.947 to 0.973), McDonald's Omega (0.950 to 0.973), AIC (0.721 to 0.859), and CR were used.

This questionnaire can be used to determine the factors associated with women's quality of life. "Postpartum quality of life assessment" addresses reproductive rights, to which other general measures do not give as much attention [12, 24, 25].

Translated to Persian version of the MAPP-QOL could be used in epidemiological and clinical postpartum studies. Those involved in postpartum care, such as midwives in health centres, are recommended to use it. Problem cases of postpartum women can be found using studies and assessments of the postpartum quality of life. Thus necessary counselling and interventions to solve problems that may occur due to the reduced postpartum quality of life of women can be provided.

### Strength

This study's strength is the cultural diversity of the people in Tehran, which combines different types of Iranian ethnicities, making the study more generalisable.

### Limitations

One of the limitations of this study was the lack of other translations of the MAPP-QOL psychometric instrument, which only made the instrument's psychometric results comparable to the original version. Besides, this instrument's number of questions seems to be high for a mother who is responsible for taking care of her baby. For this reason, it is suggested that in future studies, a short version of the instrument be designed and its psychometrical properties put under examine. Another limitation was that we could not sample from all over Iran, so we only chose participants from ten Tehran's health centres. Of course, a variety of Iran ethnicities are found in Tehran. Through further research, it can be determined whether this study's generalisation to the whole country of Iran is valid or not.

## Conclusion

Based on the results obtained, the Persian version of MAPP-QOL was found to have acceptable validity and reliability in the study population with the finalised items. Therefore, the Persian version of MAPP-QOL can be used as an appropriate tool for assessing the quality of life of postpartum women, and identifying reductions in the quality of life of women in this period so they can benefit from healthcare services. Health care services can use this tool to assess women's quality of life in the postpartum period and improve it by performing appropriate interventions in the required dimensions. For example, it can help provide psychological services to prevent postpartum depressions, and health care services to improve mothers' physical health.

## Data Availability

The datasets used and/or analysed during the current study are available via contacting the corresponding author on reasonable request.

## Abbreviations

MAPP-QOL: Maternal postpartum quality of life
QOL: Quality of life
EFA: Exploratory factor analysis
KMO: Kaiser–Meyer–Olkin index
ICC: Intra-class correlation coefficient
R2: Square of multiple correlations
SEM: Structural equation modeling
SRMR: Standardised root mean square residual
CFI: Comparative fit index
NFI: Normed fit index
GFI: Goodness of fit index
AGFI: Adjusted goodness of fit index

## Declarations

### Participate

The participants read the written consent form before filling in the questionnaire. After being introduced to the study, the postpartum women voluntarily completed the MAPP-QOL. The study objectives were explained, and participants were assured of the confidentiality of all data.
